# Narrative-Based Environmental Education Improves Environmental Awareness and Environmental Attitudes in Children Aged 6–8

**DOI:** 10.3390/ijerph19116483

**Published:** 2022-05-26

**Authors:** Ben Yang, Ningning Wu, Zepeng Tong, Yan Sun

**Affiliations:** 1 Key Laboratory of Behavioral Science, Institute of Psychology, Chinese Academy of Sciences, Beijing 100101, China; jessica_yang2008@126.com (B.Y.); 310024@hsu.edu.cn (N.W.); tongzp@psych.ac.cn (Z.T.); 2 Department of Psychology, University of Chinese Academy of Sciences, Beijing 100049, China

**Keywords:** narrative, environmental education, environmental awareness, environmental attitudes

## Abstract

Environmental education can effectively raise people’s awareness of environmental protection and encourage appropriate behaviors. This study explored the effect of narrative-based environmental education on children’s environmental awareness. To this end, we recruited first- and second-grade students from two elementary schools to participate in an experiment in which differences between the group receiving narrative-based environmental video education and the control group were compared. It was found that narrative-based environmental education can effectively promote children’s environmental awareness, which was mainly reflected in their environmental knowledge and environmental attitudes, however, not significant in their pro-environmental behavior intention. These findings support the implementation of environmental education for students in lower grades in the future.

## 1. Introduction

After the Second Industrial Revolution, increased industrial production, resource development, and personal consumption have exacerbated harmful effects on the environment [1]. Since the beginning of the 21st century, environmental problems have become increasingly apparent with global warming. According to the 2021 annual report of the United Nations Environment Programme [2], although countries around the world have made many efforts to control temperature rises, ensure biodiversity, and deal with pollution and waste, these problems are still a triple earth crisis that requires close attention in the future. After the COVID-19 pandemic in 2019, less than 20% of total recovery spending was allocated to green spending to restore the economy and rebuild homes. Due to the urgency of environmental issues, humanity must take immediate action. Some studies suggest that individuals’ incorrect environmental behavior may be due to a lack of specific environmental knowledge [3] or awareness of the need to protect the environment [4]. Using China as an example [5], the problems of people’s environmental awareness are mainly manifest in the lack of sufficient attention paid by citizens to environmental issues, as the direct living environment is emphasized over the indirect living environment; the overall level of environmental knowledge is low, and knowing and saying more but doing less indicates a serious “government dependence” and “self-protection type.” Therefore, imparting environmental knowledge and cultivating environmental awareness to the public to guide them toward the right environmental behavior could effectively improve the overall ecological environment [6].

Environmental education aims to make citizens environmentally literate with the knowledge and skills necessary to meet the environmental challenges facing the world today [7]. It can provide the public with a comprehensive understanding of the ecosystem, improve their ability to observe their surroundings and identify and solve problems, and develop methods and skills to confront environmental issues [8]. It can also form attitudes, values, and beliefs in protecting and improving the environment; enhance awareness of the urban and rural environment; and encourage people to propose individual, collective, and society-wide behavioral norms conducive to the well-being of the environment. Hence, it can be said that environmental education can guide people to develop correct environmental behavior and improve overall environmental quality resulting in a better life.

Over the past 25 years, research on the output, scope, type of design, and approach of environmental education has gained momentum [9,10,11]. In 1972, the International Union for Conservation of Nature (IUCN) suggested that environmental education should incorporate the following teaching methods: teaching in and about the environment using devices such as storytelling, problem analysis, role-playing, simulation games [12], experiments, field observations, and instant associations. Previous studies have shown that role-playing, games, and dramas can improve environmental knowledge, attitudes, and behaviors among both elementary and secondary school students [13]. In addition, countries worldwide have raised students’ environmental awareness and environmental protection skills through television, video, radio, and technology exhibitions.

In recent environmental education research, narrative methods have attracted attention. A study on climate change [14] found that story-structured narratives help achieve better personal experiences and emotional engagement, thereby promoting pro-environmental behavior. Narratives, which shed light on the characteristics of a story [15], improve learners’ understanding of, interest in, and engagement with the object of study [16]. Other studies have also noted that narratives can trigger learners’ emotions [17,18] or have a deeper cognitive [19] and behavioral [20] impact on learners. In addition, studies have found that in terms of individual memory, autobiographical memories that resonate with the theme of the story may be activated by narratives [21], and these activated memories affect past concepts [22]. Thus, narrative can be seen as a process of constructing new knowledge and experience in a more accessible way than previous knowledge and experience [23], where researchers can adopt diverse genres or tools, such as case studies, biographies [24], audio, video, and stage performances, to better communicate the messages to the audience for behavioral changes.

Currently, there are limited studies on the impact of narrative-based environmental education on children’s environmental awareness. Overall, implementing environmental education for first- and second-grade elementary students needs much improvement. Most schools do not offer environment-related courses. Since children’s environmental education plays a fundamental role in the formation of their awareness, it should be carried out on students in their early school years. Fostering children’s environmental awareness is of significant importance for a country’s future development and environmental policy formulation. This study used video as a medium because it can record an event in a three-dimensional image, present it visually with a dynamic effect, has more advantages than still pictures or audio forms, and is easy to use with children. In addition, wild animals were used as the main content, and animation elements were added to obtain the artistic effect of free movement of the image through the principle of visual afterimage, giving full play to exaggeration and fantasy that most find difficult to express [25]. Moreover, some studies have shown that watching cartoons can promote the development of children’s prosocial behavior [26], and its use in teaching can facilitate children’s understanding of relevant ideas and knowledge in a witty and humorous atmosphere while improving their thinking ability [27]. Therefore, short videos were selected as the narrative medium and then broadcast on a public platform to establish whether environmental education’s narrative content could positively impact children’s environmental awareness and offer feasible practices for environmental education.

## 2. Theoretical Background

### 2.1. Research and Application of Narrative Method

The narrative is defined as, “a representation of connected events and characters that have an identifiable structure, is bounded in space and time, and contains implicit or explicit messages about the topic being addressed.” [28]. According to this definition, a narrative is a symbolic representation of events with temporal relations or causality. Narratives can be constructed by employing a variety of media, such as written language, visual images, gestures, movements, or a combination of these media forms.

Compared with other methods, narrative methods have different characteristics. Research shows that narratives are easier to understand and more appealing to audiences than traditional logical science communications [29]. Dahlstrom [16] argued that, in essence, the use of logical science information follows deductive reasoning, while the use of narrative information follows inductive reasoning. In addition, research has shown that engaging in emotional stories and interacting with their characters [30,31], compared to using only information frameworks [32], can more effectively stimulate prosocial behavior [32,33,34]. Narratives are more persuasive than non-narratives because they increase engagement by directing the viewer into the fictional world of the story and making the audience identify with its characters [35,36]. Viewers involved in the narrative participate in the storyline and immerse themselves in the narrative process with the emotions of the characters and the course of events within [35], whether fictional or not [37]. In comparison with expository texts, narrative texts are read and recalled twice as fast, regardless of familiarity with the topic or interest in the actual content [38,39]. Glaser et al. [40] noted that compared to traditional explanatory courses, the narrative provides four factors that facilitate the acquisition of scientific knowledge: dramatization, emotionalization, personalization, and fiction. In the field of environmental protection, studies have shown that climate change issues expressed in narrative terms promote environmentally beneficial behaviors more than explanatory text [14].

Narrative is commonly used in different fields, such as medicine, sociology, history, psychology, and communication. Narrative methods have been widely applied in education in recent years. Maria do Carmo Galiazzia et al. [41] discussed the effective employment of narratives in learning groups focusing on environmental education. Morris et al. [14] hypothesized that in terms of discussions about climate change, narratives send visual and audio information to the brain, and the narrative content can facilitate experiential processing, making people empathize with the story and inducing the brain to send action commands that prompt pro-environmental behavior. This hypothesis was confirmed. Research outside China on narrative-based education began in the 1980s, with Canadian and North American scholars as major contributors producing notable results. In China, the narrative method was only introduced into educational research in the late 1990s. In particular, after 2000, results in narrative-based education have been studied [42]. Many studies have focused on children’s education; however, these are sporadic and unsystematic. Currently, as the Internet and social media are widely employed in education, the new media landscape is changing the way science is communicated to the public [31]. According to the National Science Board [43] once school education is completed the public receives most scientific information via mass media. Narratives in visual media present information in both auditory and visual ways, making them easier to remember, thus facilitating guidance for future behavior [44].

### 2.2. Research and Factors of Environmental Awareness

Modern environmental awareness originated in the West. In 1968, Roth, an American scholar, first proposed the concept of environmental literacy, whose meaning is close to that of environmental consciousness [45], and tried to improve citizens’ environmental literacy and solve the problem of environmental illiteracy through environmental education [46]. In addition, wording such as the new environmental paradigm [47] and environmental concern [48] are often used when discussing environmental awareness. Although there are some differences in meaning, they all have a common core, that is, they reflect people’s views on the relationship between man and nature. At present, there is no unified definition of the connotation of environmental awareness. Dunlap and Jones pointed out in “Environmental Concern” that there are probably hundreds of definitions of environmental awareness [49]; even in China, more than 30 definitions of environmental awareness can be found in books and periodicals [50]. After analysis, there are two main views, one of which is represented by Hong Dayong’s research, which states that environmental awareness should include four links: environmental knowledge, values, protection attitudes, and protection behaviors, which are interlocking and layered [51]. In the West, early research also regarded environmental protection behavior as part of environmental awareness, but with the deepening of research, another view has gradually reached consensus in the academic community, that is, environmental behavior should exist independently of environmental awareness as a separate variable because the latter is mainly to study the extent to which it can change the former. If we conceptually regard environmental protection behavior as part of environmental awareness, it becomes difficult to explain the relationship between the two [52].

The indicator systems proposed by Western scholars to measure environmental awareness can be primarily divided into three categories: first, the ecological attitudes and knowledge scale proposed by Maloney and Ward in 1973; second, the new ecological paradigm (NEP) scale proposed by Dunlap et al. in 1978; third, the environmental awareness scale proposed by German scholars Urban, Schahn, and Diekmann Preisendorfer in the 1990s. Maloney and Ward [53] believed that environmental awareness is the attitude of human interaction with the environment, including three aspects: cognition, emotion, and impulse. Initially consisting of 130 items, the scale was divided into four subscales: affect (34 items), knowledge (24 items), verbal commitment (36 items), and actual commitment (36 items). In a subsequent study, the scale was reduced to 45 items [54]. In the late 1970s, a paradigm differing from the mainstream in Western industrialized countries was proposed and named the new ecological paradigm [55]. Originally, with 12 items [47], in 2000 the scale was expanded to 15 [56]. German scholar Dieter Urban [57] believed that environmental awareness should include three relatively independent dimensions: environmental values, environmental attitudes, and intention to act ecologically responsibly, with environmental attitudes as the core part. From the three most influential scales in American and European academia, we find the following consensus: first, environmental attitudes are the most basic components of environmental awareness; second, depending on the purpose of the study, environmental values or behavioral intention in the environment can be regarded as a part of environmental awareness; and finally, environmental knowledge can become the third part of environmental awareness. Therefore, based on the characteristics of children’s cognitive development, this study selected environmental knowledge, environmental attitudes, and pro-environmental behavioral intention to measure children’s environmental awareness.

Several factors affect environmental awareness. From the perspective of individuals [58], it is determined by factors such as childhood experience, gender, age, values, educational background, sense of responsibility, cognitive differences, and regions. In the context of a whole society, citizens’ environmental awareness is affected by religious beliefs, culture, ethnic differences, urban–rural differences, social norms, social class, environmental issues [58], prevailing values, the degree of mass media penetration, the promotion of environmental education, and environmental protection efforts, among others. In general, social and economic development, the educational background of citizens, and social and political factors are closely linked to the level of environmental awareness. Therefore, it is imperative to adopt measures for its improvement.

## 3. Studies

This study is divided into two experiments, implemented in schools and families. Considering that both school and family education are important components of children’s environmental education, the results can provide a reference for both. Thus, Experiment 1 was implemented by teachers in the classroom, and Experiment 2 was by children’s parents. However, the answers to the questionnaires were collected in school classrooms, and the teacher read the questionnaire instructions and collected the questionnaires after the children had answered. The experimental materials were the same video material and the same set of scales, including the children’s environmental knowledge scale, children’s environmental attitude scale, and children’s pro-environmental behavior intention scale. The experimental materials were short videos made by the research team called *Lessons for Curious Kids* (refer to Table A1 in Appendix A for the specific content of the experimental materials for each lesson). The video has been posted on the “The New media of Chinese Academy of Sciences” official account, and the video viewing link is https://www.cas.cn/newmedia/dsp/202009/t20200916_4760034.shtml (accessed on 15 September 2020). The following describes the specific contents of the three scales.

The children’s environmental knowledge scale refers to the Chinese version of the environmental knowledge scale, CEKS [59], which contains ten items (see Table A2 in Appendix B for details), all of which are true or false questions, some of which are related to the experimental materials. Given the limitations of the students’ cognition, all questions were marked with Chinese phonetic alphabet for the students’ convenience. A correct answer was allocated 1 point, and an error 0. The results for all the items were summed and averaged. The larger the value, the higher the level of the individual’s environmental knowledge. The instructions and sample questions were as follows.

*Children, please read the following sentences carefully; draw a tick in parentheses (√) after the sentence you think is correct, and a cross in parentheses (* *×) after the sentence you think is wrong.*

*1.*  *Weather changes will not affect our lives.*
*2.*  *Wild animals carry viruses, bacteria, and parasites.*

The children’s environmental attitude scale is based on the research and literature of scholars outside China, combined with conference documents related to sustainable development in China and children’s cognitive development stages. When the initial version was roughly confirmed, the researcher finalized items included in the questionnaire after many rounds of discussions with the instructor and several postgraduates in children’s education, with numerous revisions and improvements to the questions and expressions. Each question was marked with a Chinese phonetic alphabet. Finally, after the pre-experiment of the children’s environmental attitude scale, it was found that the children understood the questions well, and thus the final version was confirmed. Cronbach’s α coefficient of the questionnaire was 0.790, indicating its high reliability. To help children better understand the meaning of the questions, the scale adopted emojis to show the degree of intention. There was a total of ten reverse-scored questions (see Table A4 in Appendix B for details), with nine emojis in total (four crying faces, one without expression, four smiley faces) scored at nine points, that is, the largest crying face scored nine points, and the largest smiley face scored one point. The results for all the items were summed and averaged. The larger the value, the stronger the individual’s environmental attitude. The teacher ensured that the children understood the questions. The instructions and sample questions were as follows.

*Children, there are nine emojis following each sentence. The four crying faces on the left indicate sadness. A bigger crying face shows greater sadness. The four smiling faces on the right indicate happiness. A bigger smiling face shows greater happiness. The emoji in the middle is neither crying nor laughing, indicating neutral emotions. After reading each sentence, please choose a smiling face if you are happy, and a crying face if you are sad. The degree of happiness or sadness is up to you. If you are neither happy nor sad, choose the emoji in the middle. Please tick under the expression you choose.*

*1.*  *The factory discharges waste into the river.*
*2.*  *Using pangolin scales to make traditional Chinese medicine.*

The children’s pro-environmental behavior intention scale is a revision of the environmental behavior scale designed by Stern and Guagnano [60] and includes 15 multiple-choice questions (see Table A3 in Appendix B for details). Each question was marked with a Chinese phonetic alphabet. After the pre-experiment, Cronbach’s α coefficient of the scale was 0.760. To facilitate the answering process, the questions were followed by circles to indicate the degree of intention. The larger the circle, the greater the pro-environmental behavioral intention of the children. There were a total of nine circles with nine points for scoring; that is, the largest circle was scored at nine points and the smallest circle one point. Among them, “*seeing the beautiful flowers in the park, picking them, and bringing them home*”, and “*Taking many meals at one time and leaving food behind on the plate that you cannot finish*”. All the points were summed and averaged. The larger the value, the stronger the individual’s intention to engage in pro-environmental behavior. The teacher made sure that the children understood the questions. The instructions and sample questions were as follows.

*Please read the questions carefully. Each of the following sentences describes a behavior, and there are nine circles under each sentence. From left to right, the circles get larger. Please read each behavior carefully. A larger circle suggests greater intention to follow the described behavior, please tick the circle accordingly.*

*1.*  *Going to the supermarket with own shopping bags.*
*2.*  *Observing small animals when going outdoors.*

### 3.1. Study 1

Study 1 aimed to measure the effectiveness of narrative-based environmental education on children’s environmental knowledge, pro-environmental behavior intention, and environmental attitudes.

#### 3.1.1. Participants

There were 143 student participants (*N* = 143) from all four classes in the second grade of elementary school. The composition of the participants is presented in Table 1.

#### 3.1.2. Procedure

The experiments were conducted at school. Participants were divided into narrative and control groups. The students in the narrative group received normal teaching at school and watched seven short environmental education videos, while the students in the control group only received normal teaching without watching the videos.

#### 3.1.3. Results

Employing SPSS version 22.0 for data analysis, this study adopted descriptive statistics, reliability tests, and independent samples *t*-tests [61]. Table 2 shows the descriptive statistics of the indicators of children’s environmental awareness and the independent samples *t*-test results.

Table 2 shows that the environmental knowledge score of the narrative group (M = 7.638, SD = 1.533) was significantly higher than that of the control group (M = 6.892, SD = 1.575), and there was a significant difference in the scores of the two groups (*t* (143) = 2.849, *p* = 0.005 < 0.01); the pro-environmental behavior intention score between the narrative group (M = 7.936, SD = 0.943) and the control group (M = 7.913, SD = 1.055) did not differ significantly (*t* (143) = 0.141, *p* = 0.888); and the environmental attitude score between the narrative group (M = 8.368, SD = 0.956) and the control group (M = 8.123, SD = 1.016) did not significantly differ (*t* (143) = 1.483, *p* = 0.140).

#### 3.1.4. Discussion

The data from Study 1 showed that after the intervention, students in the narrative group had better environmental awareness, of which environmental knowledge increased significantly. Although their pro-environmental behavioral intention and environmental attitudes improved, there was no statistical significance. The data show that the video-based narrative method can effectively improve children’s environmental awareness, but only their environmental knowledge. This educational method does not promote environmental attitudes and pro-environmental behaviors within a short period of time.

### 3.2. Study 2

To further explore whether narrative-based environmental education has a long-term impact on children’s environmental awareness, we improved the experimental process of Study 1. In Study 2, the learning process was integrated into family education, during which the teacher sent environmental education videos to parents, who in turn instructed their children. As a result, parents implemented environmental education. In addition, relevant studies have shown that the duration of education has a significant impact on its effectiveness. In Study 1, seven environmental education videos were watched in one period, for a total of 13 min. Together, they presented too much knowledge to second-grade children, making it difficult for them to memorize unfamiliar environmental knowledge. These children also had short attention spans, which led to errors. Therefore, taking into consideration the nature and features of children’s learning processes, Study 2 sent one environmental education video to parents each day for seven days in a row, asking the parents to play them for their children. None of the lessons included overwhelming environmental knowledge and thus did not impose a burden on the children’s memory.

#### 3.2.1. Participants

The participants were students in the first and second grades of another elementary school. A total of 159 students participated in the pre-test, post-test, and long-term effect tests. The composition of the participants is presented in Table 3.

#### 3.2.2. Procedure

Participants were divided into narrative and control groups. The former watched short videos at home, while the control group did not watch any. After seven days of observation, the participants completed the questionnaire and questions in the classroom.

Step 1: On Monday, students completed the paper questionnaire as a pre-test. The class teachers helped them read the questions, and the test took approximately 20 min. The questionnaires were collected after completion.

Step 2: From Monday to Sunday, the class teachers sent a 1- to 3-min video to the class group chats every day, asking parents to play it to the students. Parents were required to take photographs of the learning process and send them to the group chat.

Step 3: After a week of video learning, the students completed the paper questionnaire as a post-test. The class teacher helped the students read the questions, and the test took approximately 20 min. The questionnaires were collected after completion.

Step 4: One week after the post-test, the students completed another test using paper-based questionnaires. The class teacher helped the students read the questions, and the test took approximately 20 min. The questionnaires were collected after completion.

Paper questionnaires were distributed offline. A total of 181 questionnaires were collected in the pre-test, 179 in the post-test, and 178 in the long-term effects test. Using a principal component analysis, 159 valid questionnaires were selected.

#### 3.2.3. Results

This study adopted SPSS version 22.0 for data analysis, mainly using descriptive statistics, reliability tests, Pearson correlation analysis, two-way repeated-measures analysis of variance (ANOVA), and independent samples *t*-tests [61]. The results are as follows. Table 4 shows the descriptive statistics of various indicators of children’s environmental awareness.

As shown in Table 5, there was no significant difference in the environmental knowledge, pro-environmental behavioral intention, or environmental attitudes of the students between the control and narrative groups during the pre-test. This variable in Study 2 was effectively controlled, meaning that their experience and knowledge did not affect the experiment.

**Figure 1:**
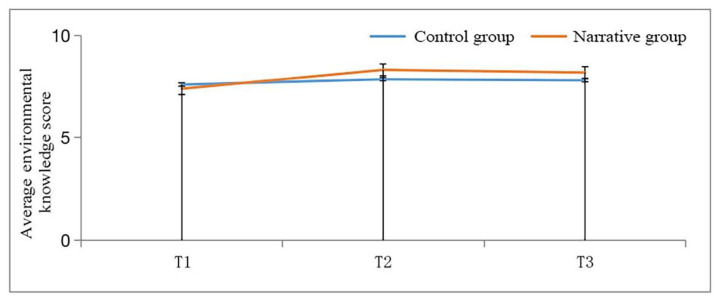
Two-way repeated-measures ANOVA of children’s environmental knowledge.

A two-way repeated-measures ANOVA verified the influence of narrative-based environmental education on various dimensions of children’s environmental awareness and explored the long-term impact of such education. The results of environmental knowledge, attitudes, and pro-environmental behavioral intentions are reported separately below. The results for environmental knowledge are presented in Figure 1 and Table 6.

Figure 1 and Table 6 show that the main effect of environmental knowledge on the education method is significant (F (1, 157) = 5.506, *p* < 0.01), indicating that the acquisition of environmental knowledge in the narrative group was higher than that in the control group. The main effect of time was also significant (F (1, 157) = 12.466, *p* < 0.01), which means that the knowledge retention effect after one week of study was higher than that before the experiment. The interaction between time and educational method was significant (F (1, 157) = 5.908, *p* < 0.01), indicating that knowledge retention was better in the narrative group.

Simple effects analysis showed that the level of environmental knowledge in the narrative group before the intervention (T1) (M = 7.360, SD = 1.674) was significantly different from that after the intervention (T2) (M = 8.310, SD = 1.285) (*p* < 0.01), indicating that their environmental knowledge improved significantly after the intervention. However, there was no significant difference between T2 (M = 8.310, SD = 1.285) and the test after one week (T3) (M = 8.180, SD = 1.346) (*p* > 0.05), which means that knowledge can be retained for some time after the intervention. The level of environmental knowledge between T1 (M = 7.360, SD = 1.674) and T3 (M = 8.180, SD = 1.346) was significantly different (*p* < 0.01). In T3, the level of environmental knowledge was still higher than before the intervention.

**Figure 2:**
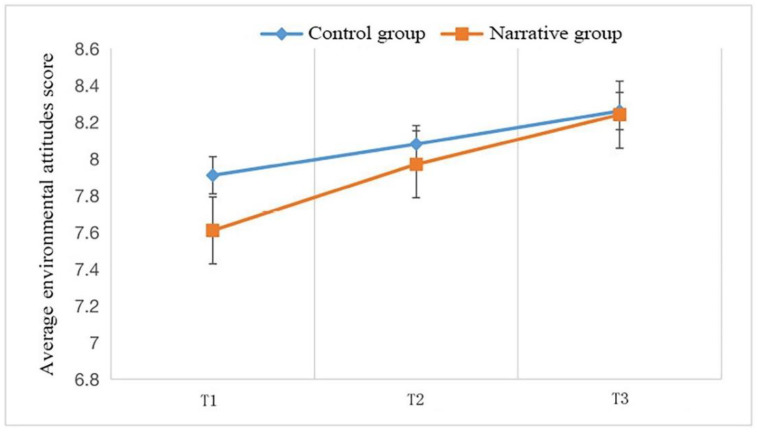
Two-way repeated-measures ANOVA of children’s environmental attitudes.

Figure 2 and Table 7 show the results of the two-factor repeated-measures ANOVA for children’s environmental attitudes. The results showed that the main effect of environmental attitudes on the education method was significant (F (1, 157) = 3.431, *p* < 0.05), which shows that the improvement of environmental attitudes in the narrative group was higher than that in the control group. The main effect of time was also significant (F (1, 157) = 7.528, *p* < 0.01), which means that environmental attitudes significantly improved after one week of intervention. The interaction between time and education method was significant (F (1, 157) = 3.182, *p* < 0.05), indicating that the impact of education method on environmental attitudes increased over time.

The simple effects analysis showed that there was a statistically significant difference (*p* < 0.01) in the level of environmental attitudes in the narrative group between T1 (M = 7.622, SD = 1.122) and T2 (M = 7.965, SD = 1.081), which proves that the level of environmental attitudes significantly improved after the intervention. The difference between T2 (M = 7.965, SD = 1.081) and T3 (M = 8.242, SD = 0.908) in environmental attitudes was statistically significant (*p* < 0.01), demonstrating that environmental attitudes still improved one week after the intervention ended. The improvement in environmental attitudes between T3 (M = 8.242, SD = 0.908) and T1 (M = 7.622, SD =1.122) was statistically significant (*p* < 0.01), suggesting that narrative-based environmental education had a lasting impact on the improvement of environmental attitudes.

**Figure 3:**
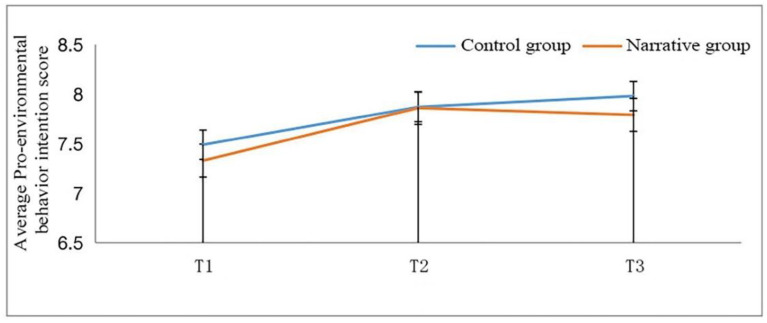
Two-factor repeated-measures ANOVA of children’s pro-environmental behavior intention.

Figure 3 and Table 8 show the results of the two-factor repeated-measures ANOVA of children’s pro-environmental behavior intention. The results show that there is no significant difference in the main effect of pro-environmental behavior intention in the education method (F (1, 157) = 0.994, *p* > 0.05), indicating that there was no difference in influencing pro-environmental behavior intention of the narrative-based education compared with the traditional teaching method. The main effect of time was significant (F (1, 157) = 20.161, *p* < 0.001), which means there is an overall upward trajectory in students’ pro-environmental behavior intention as time passes. The interaction between time and education method was not significant (F (1, 157) =1.106, *p* > 0.05), suggesting that narrative-based education cannot promote pro-environmental behavioral intention within a short period.

A simple effects analysis showed that the pro-environmental behavioral intention of the narrative group in T1 (M = 7.332, SD = 1.161) was significantly different from that in T2 (M = 7.86, SD = 1.138) (*p*< 0.01), which means that the intervention improved pro-environmental behavioral intentions. There was no statistical difference in pro-environmental behavioral intention between T2 (M = 7.86, SD = 1.138) and T3 (M = 7.79, SD = 1.261) (*p* > 0.05), indicating that, after the intervention, pro-environmental behavior intention gradually decreased. The level of pro-environmental intention in T3 (M = 7.79, SD = 1.261) was significantly different from that in T1 (M = 7.332, SD = 1.161) (*p* < 0.01), indicating that although pro-environmental intention decreased one week after the intervention ended, it was still improved in comparison with that before video learning.

The independent sample *t*-tests of gender and age showed that there was no significant difference between gender in environmental knowledge, environmental attitudes, or pro-environmental behavior (*p* > 0.05). In terms of grades, second-grade students had significantly better environmental knowledge and attitudes than their first-grade counterparts (*p* < 0.05). The difference was verified in all three tests, but no significance was found in pro-environmental behavior between grades (*p* > 0.05).

#### 3.2.4. Discussion

Data analysis showed that children’s environmental knowledge and attitudes significantly improved after the intervention, and the improvement did not weaken over time. Children’s pro-environmental behavioral intention did not differ significantly among the pre-test, post-test, and long-term effect tests. The results show that narrative-based environmental education can effectively improve children’s environmental knowledge and attitudes under a certain duration of intervention, while the improvement in pro-environmental behavior intention is not significant.

## 4. Discussion

The following is a discussion of the three indicators.

The promotion of environmental knowledge plays a vital role in increasing environmental awareness. Achieving educational goals requires both appropriate program content and teaching strategies. The narrative method in this study accommodated the cognitive features of first- and second-grade students by integrating role-playing and dialogue in the narrative process to enhance their understanding of environmental protection at the cognitive, emotional, and behavioral levels. In addition, selecting videos for the narrative-based education in this study is suitable for today’s educational reality. Previous studies have also pointed out that the use of videos for environmental education has a positive impact on teaching in various dimensions such as knowledge understanding and emotional expression [62]. The teaching duration is also a factor that affects the effectiveness of environmental education. Sellmann and Bogner [63] argued that longer interventions may contribute to better knowledge gain. This is supported by Stern et al. [64], who compared the effects of three- and five-day educational interventions in national parks and found that longer interventions led to better cognitive outcomes. In addition, in a four-day environmental education course on water issues, a test conducted one month later found that the students had significant improvements in knowledge acquisition and their understanding of how to act and do so more effectively [65]. In this study, the duration of the educational interventions in Study 1 and Study 2 was one day and seven days, respectively. The second intervention fully demonstrated the effect of a longer duration on environmental knowledge acquisition.

The results show that narrative-based environmental education cannot form children’s environmental attitudes within a short period. Improvement in environmental attitudes is only possible under long-term education. According to the theory of attitude formation [66], Kelman proposed a three-stage theory of attitude formation: compliance, identification, and internalization. In the early stages of life, attitudes are largely formed through compliance. Internalization means that an individual truly believes and accepts the views of others from the heart, making them an integral part of their attitude structure. As attitudes are stable and consistent, their formation always takes a considerable amount of time. People are not born with attitudes, they are formed in the process of socializing themselves in the later stages of their life. In Study 1, the children completed the environmental awareness questionnaire immediately after the narrative-based environmental education, which lasted for only one day. It was impossible to change children’s environmental attitudes within a short period of time. Therefore, improvement in environmental attitudes was not significant. Bogner [67] conducted a one-week outdoor learning experiment involving students in a public park to explore whether the study duration had an impact on students’ environmental knowledge and attitudes. The study found that a one-week course had a significant positive effect on students’ environmental knowledge and attitudes, and the effects lingered even a month later. Relevant research [68] has shown that compared with one-day programs, continuous multi-day courses have superior results in students’ knowledge acquisition. Therefore, the seven-day environmental education program in Study 2 improved both children’s environmental knowledge and attitudes.

The results of Study 1 and Study 2 show that the effect of narrative-based environmental education on children’s pro-environmental behavioral intention is not significant. According to the social learning theory proposed by Bandura [69], children’s prosocial behavior is acquired by observing and imitating the behaviors of different people in social life and, therefore, is a process largely conditioned by social factors. Prosocial behavior is formed through continuous learning, which is referred to as the process of socialization. The formation of pro-environmental behavior also takes time. Therefore, it is difficult to improve children’s pro-environmental behavior in a short period, such as with one-day or seven-day instructions. In addition, according to the theory of embodied cognition, cognitive processes are rooted in the interaction between the human body and the world [70]. Therefore, knowledge based only on real contexts is vivid for learners. Knowledge acquisition is possible by engaging in activities in real-life situations. In this study, although children’s environmental knowledge increased, it was difficult to translate what they had learned into their daily behavior because of their lack of first-hand experience. According to reinforcement theory [71], when children’s pro-environmental behavior is not positively reinforced, it diminishes or even vanishes.

The theoretical contribution and practical guidance of this paper are as follows: Although the narrative method has been widely applied in the field of education and has received much attention in environmental education research, few applied studies have researched the lower grades of elementary schools. Environmental education should cover the entire age group, and this study fills the gap in the research on this age group. In the past, environmental education was mainly implemented by teachers. This study investigated not only the school education scene but also the family education scene. The experimental results provide effective methods and suggestions for schools and families to carry out environmental education. Combined with the reality in China, the status of environmental education is still marginalized. Most schools do not offer special environmental education courses, and most of the teaching methods are dictated by teachers or are close to nature. The narrative method adopted in this study can provide an additional reference for teachers.

## 5. Conclusions

The main conclusions are as follows. This study proves that narrative-based environmental education can have a positive impact on children’s environmental awareness. Compared with traditional teaching methods, narrative-based education can deepen children’s understanding of environmental knowledge and increase their attention to environmental issues, which is conducive to the connection between children and nature, as it teaches them to love nature and care for the environment. Since behavioral change is a slow process, narrative environmental education should be carried out continuously in elementary and secondary schools to promote the intention of pro-environmental behavior. It should be integrated into classroom activities and extracurricular practice to stimulate children’s emotions toward the natural environment, resulting in a benign interaction with the environment, and promoting changes in behavioral habits.

## 6. Limitations and Future Studies

The limitations of this research lie in the small sample sizes of participants, who were recruited from only two elementary schools in Huangshan City, China, and thus the samples are not sufficiently representative, which limits the general applicability of the results to a certain extent. In the future, stratified sampling according to demographic factors, such as regions, schools, grades, ages, and gender, should be undertaken to gain a deeper understanding of the topic concerned and improve the overall validity of the research.

In follow-up research, it is recommended to explore whether students’ improved environmental awareness will influence their parents’ environmental awareness or environmental behavior. Teachers’ environmental values should also be considered. Researchers could also compare models of one-to-one parent–child interactive education and one-to-many teacher–student interactive education. In the future, when applying the narrative teaching method, other media forms such as audio and drama plays can be considered to explore whether there is a better medium that more effectively imparts knowledge. Narrative thinking and empirical thinking would also make good topics for discussion. While narrative thinking is based on knowledge and experience, empirical is based on principles and logic. How to impart scientific knowledge through narrative in teaching is a direction worthy of research.

## Figures and Tables

**Table 1:** Composition of participants (*N* = 143).

Groups	Class One	Class Two	Class Three	Class Four	Total
	*N*	*N*	*N*	*N*	
Narrative group		33	36		69
Control group	37			37	74

**Table 2:** The descriptive statistics of the indicators of children’s environmental awareness and the independent samples *t*-test results.

Indicators	Groups	M	SD	*t* Value	*p* Value
Environmental knowledge	Narrative group	7.638	1.553	2.849	0.005 **
	Control group	6.892	1.575		
Pro-environmental behavior intention	Narrative group	7.936	0.943	0.141	0.888
	Control group	7.913	1.055		
Environmental attitudes	Narrative group	8.368	0.956	1.483	0.140
	Control group	8.123	1.016		

(Note: **. Significance at the 0.01 level (two-sided)).

**Table 3:** Composition of participants (*N* = 159).

Groups	Class (1) Grade One	Class (2) Grade One	Class (1) Grade Two	Class (2) Grade Two	Total
	*N*	*N*	*N*	*N*	
Narrative group	35		40		75
Control group		45		39	84

**Table 4:** Descriptive statistics of various indicators of children’s environmental awareness.

	T1 (M ± SD)	T2 (M ± SD)	T3 (M ± SD)
Environmental knowledge	7.49 ± 1.492	8.04 ± 1.268	7.99 ± 1.295
Pro-environmental behavior intention	7.42 ± 1.16	7.83 ± 1.10	7.89 ± 1.210
Environmental attitudes	7.78 ± 1.03	8.03 ± 0.99	8.25 ± 0.88

Notes: T1, pre-test; T2, post-test; T3, long-term effect test.

**Table 5:** The results of independent samples t-test of children’s environmental awareness in the experimental group and the control group.

	Environmental Knowledge	Pro-Environmental Behavior Intention	Environmental Attitudes
Control group	7.610 ± 1.305	7.498 ± 1.162	7.910 ± 0.935
Narrative group	7.360 ± 1.674	7.332 ± 1.161	7.622 ± 1.122
*t*	1.071	0.895	1.747
*p*	0.286	0.372	0.083

Notes: *p* > 0.05. Table 5 shows the independent samples *t*-test results for each indicator of environmental awareness in the narrative and control groups during the pre-test (T1).

**Table 6:** Two-way repeated-measures ANOVA of children’s environmental knowledge.

	T1 (M ± SD)	T2 (M ± SD)	T3 (M ± SD)	F Value	*p* Value
Control group	7.610 ± 1.305	7.850 ± 1.233	7.800 ± 1.267	5.506	0.006
Narrative group	7.360 ± 1.674	8.310 ± 1.285	8.180 ± 1.346		

**Table 7:** Two-way repeated-measures ANOVA of children’s environmental attitudes.

	T1 (M ± SD)	T2 (M ± SD)	T3 (M ± SD)	F Value	*p* Value
Control group	7.910 ± 0.935	8.082 ± 0.924	8.256 ± 0.890	3.431	0.036
Narrative group	7.622 ± 1.122	7.965 ± 1.081	8.242 ± 0.908		

**Table 8:** Two-factor repeated-measures ANOVA of children’s pro-environmental behavior intention.

	T1 (M ± SD)	T2 (M ± SD)	T3 (M ± SD)	F Value	*p* Value
Control group	7.498 ± 1.162	7.87 ± 1.070	7.98 ± 1.161	0.994	0.363
Narrative group	7.332 ± 1.161	7.86 ± 1.138	7.79 ± 1.261		

## Data Availability

All data were uploaded on the Figshare. Other researchers can download the dataset at https://figshare.com/s/f68dc8ab5bcaacf12a3c (accessed on 10 March 2020).

## Appendix A

**Table A1:** The content of experimental materials for each lesson.

Topic	Video Length	Background Music	Psychological Knowledge	Environmental Knowledge
1 What are wild animals?	2:45	Absolute music	Children at the age of 2 to 6 or 7 see the world from their perspectives. They lack the ability for independent judgment and analysis as well as external exploration. They may encounter many vague concepts. How to make children at this stage understand what are wild animals? It is important to stimulate their interest and attention by starting with clever questions.	Wild animals have not been domesticated. They do not need our care because they will find food and water on their own.
2 Who touched its ivory?	3:02	May I See U Again-MT1990	Compassion is a social emotion that refers to an individual’s ability to think in others’ shoes and feel their emotions. Compassion is not yet mature in infancy and develops gradually in early childhood.	Elephants are the largest mammals on land in modern times and ivory is an important tool for elephants to survive.
3 Are wolves bad animals?	3:35	The Forest Show	Babies understand and explore the world by seeing, tasting, touching, hearing, and taking. As they grow older, they master language and imagination, learn to think and communicate, and in adolescence, they can understand the complex logical relationship of things.	Wolves eat sheep while controlling the number of sheep. Animals are not inherently bad or good. They just have different roles to play in nature. Protecting wild animals helps maintain the ecological balance of the earth.
4 What is the relationship between wild animals and humans?	1:48	The Forest Show	Relationships refer to the state of interaction and mutual influence between things. From birth, babies explore the world through seeing, listening, and establishing relationships with others. With an in-depth exploration of their surroundings, children gradually understand different relationships.	Humans draw their inspiration for inventions from wild animals, which may carry viruses, bacteria, and parasites that threaten human health.
5 Why do some people eat wild animals?	1:52	Absolute music	There are different psychological needs behind people’s consumption of wild animals. Psychological needs are developed from physical ones and then exist independently, becoming the goal of satisfaction.	Wild animals do not have higher nutritional value. It is illegal to eat wild animals and there is a risk of disease transmission when consuming wild animals indiscriminately.
6 Why do humans get sick by eating wild animals?	1:32	Demier Vol-Raphael Beau	There are significant age-based differences in children’s cognition of disease concepts. Restricted by their thinking development, children tend to master disease concepts through concrete images.	Wild animals living in nature carry many viruses. Randomly eating wild animals will damage human health, so we should keep a certain distance from wild animals. Every wild animal is indispensable in the super-large biological chain of nature, so everyone should protect all wildlife.
7 Why do we protect wildlife	1:30	Floating	The human’s understanding of the world depends on the understanding of causal connections between things. Modern psychologist Liu Fan believes that environmental education and cultural influences play an extremely important role in the development of children’s cognitive abilities.	Although humans are at the top of the food chain and may seem powerful, we cannot survive without wildlife.

## Appendix B

**Table A2:** The scale of children’s environmental knowledge.

Number	Scoring	Items
1	These are true-or-false questions. One point for a correct answer, and zero points for a wrong answer.	Trees can resist wind and sand
2		Excessive use of fertilizers and pesticides can damage the environment
3		Weather changes will not affect our lives
4		Reducing the use of disposable tableware can save the environment
5		Car exhaust does not pollute the air
6		Domestic puppies are wild animals
7		Elephants are the largest mammals on land
8		Wild animals carry viruses, bacteria, and parasites
9		Wild animals need human care
10		Humans killing all wolves helps sheep survive better

**Table A3:** The scale of children’s pro-environmental behavior intention.

Number	Scoring	Items
1	9 circles with incremental sizes for 9-point scoring	Picking up the rubbish on the ground and throwing it into the trash can
2		Seeing beautiful flowers in the park, picking them, and bringing them home
3		Saving water when washing hands
4		Going to the supermarket with self-brought shopping bags
5		Taking a lot of meals at one time and leaving them on the plate if not finishing them
6		Discussing saving electricity and water with mom and dad at home
7		Discussing saving electricity and water with teachers and classmates at school
8		Stopping classmates or family members from picking flowers in the park
9		Visiting the zoo
10		Watching wildlife-related videos or TV shows
11		Observing small animals when going outdoors
12		Reading books or picture books about wildlife
13		Discussing wildlife conservation with mom and dad at home
14		Discussing wildlife protection with teachers and classmates at school
15		Telling classmates or family members that it is wrong to hurt wildlife

**Table A4:** The scale of children’s environmental attitudes.

Number	Scoring	Items
1	There are 9 emojis of crying faces and smiling faces for 9-point scoring	The factory discharges waste into the river
2		Cutting down trees
3		Rising temperatures cause glaciers to melt
4		Oil leaks into the sea
5		Garbage is dumped in the open air
6		Using pangolin scales to make traditional Chinese medicine
7		Making bracelets out of ivory
8		Making coats out of tiger skin
9		Making wallets out of crocodile skin
10		Melting glaciers affect polar bears’ lives

## References

[1] Th⊘gersen J. (2009). The Motivational Roots of Norms for Environmentally Responsible Behavior. Basic Appl. Soc. Psychol..

[2] Annual Report 2021 of UNEP. https://www.unep.org/zh-hans/resources/2021niandubaogao.

[3] Tanner C., Kast S.W. (2003). Promoting sustainable consumption: Determinants of green purchases by Swiss consumers. Psychol. Mark..

[4] Liu A., Osewe M., Wang H., Xiong H. (2020). Rural Residents’ Awareness of Environmental Protection and Waste Classification Behavior in Jiangsu, China: An Empirical Analysis. Int. J. Environ. Res. Public Health.

[5] Environmental Behaviors among Chinese Citizens Survey Report 2021. http://www.prcee.org/zyhd/202112/t20211225_965281.html.

[6] Ardoin N.M., Bowers A.W., Gaillard E. (2020). Environmental education outcomes for conservation: A systematic review. Biol. Conserv..

[7] Vasconcelos C. (2012). Teaching Environmental Education through PBL: Evaluation of a Teaching Intervention Program. Res. Sci. Educ..

[8] Guidelines on Implementing Environmental Education in Primary and Secondary Schools. http://www.moe.gov.cn/srcsite/A06/s7053/200310/t20031013_181773.html.

[9] Ardoin N.M., Clark C., Kelsey E. (2013). An exploration of future trends in environmental education research. Environ. Educ. Res..

[10] Rickinson M. (2001). Learners and learning in environmental education: A critical review of the evidence. Environ. Educ. Res..

[11] Stevenson R.B., Brody M., Dillon J., Wals A.E.J., Stevenson R.B., Brody M., Dillon J., Wals A.E.J. (2013). Introduction: An Orientation to Environmental Education and the Handbook. International Handbook of Environmental Education Research.

[12] Dickinson J.L., Crain R., Yalowitz S., Cherry T.M. (2013). How Framing Climate Change Influences Citizen Scientists’ Intentions to Do Something about It. J. Environ. Educ..

[13] Curtis D.J., Howden M., Curtis F., McColm I., Scrine J., Blomfield T., Ryan T. (2013). Drama and environment: Joining forces to engage children and young people in environmental education. Aust. J. Environ. Educ..

[14] Morris B.S., Chrysochou P., Christensen J.D., Orquin J.L., Barraza J., Zak P.J., Mitkidis P. (2019). Stories vs. facts: Triggering emotion and action-taking on climate change. Clim. Change.

[15] Morgan M.S. (2017). Narrative ordering and explanation. Stud. Hist. Philos. Sci..

[16] Dahlstrom M.F. (2014). Using narratives and storytelling to communicate science with nonexpert audiences. Proc. Natl. Acad. Sci. USA.

[17] Gerrig R.J. (2018). Experiencing Narrative Worlds: On the Psychological Activities of Reading.

[18] Oatley K. (1999). Why Fiction May Be Twice as True as Fact: Fiction as Cognitive and Emotional Simulation. Rev. Gen. Psychol..

[19] Green M.C., Brock T.C. (2000). The Role of Transportation in the Persuasiveness of Public Narratives. J. Personal. Soc. Psychol..

[20] Diekman A.B., Gardner W.L., McDonald M. (2000). Love Means Never Having to Be Careful: The Relationship Between Reading Romance Novels and Safe Sex Behavior. Psychol. Women Q..

[21] Mar R.A. (2004). The neuropsychology of narrative: Story comprehension, story production and their interrelation. Neuropsychologia.

[22] Strange J.J., Leung C.C. (1999). How Anecdotal Accounts in News and in Fiction Can Influence Judgments of a Social Problem’s Urgency, Causes, and Cures. Personal. Soc. Psychol. Bull..

[23] Pachler N., Daly C. (2009). Narrative and learning with Web 2.0 technologies: Towards a research agenda. J. Comput. Assist. Learn..

[24] Goodson I.F. (1994). Studying the teacher’s life and work. Teach. Teach. Educ..

[25] Zhou Q. (2009). Research on Education Value of Domestic Children’s Cartoon in Nowadays. Master’s Thesis.

[26] Su Z. (2012). An Intervention Study on Children’s Four Typical Prosocial Behavior with Animation. Master’s Thesis.

[27] Rule A.C., Auge J. (2005). Using humorous cartoons to teach mineral and rock concepts in sixth grade science class. J. Geosci. Educ..

[28] Kreuter M.W., Green M.C., Cappella J.N., Slater M.D., Wise M.E., Storey D., Woolley S. (2007). Narrative communication in cancer prevention and control: A framework to guide research and application. Ann. Behav. Med..

[29] Green M.C. (2006). Narratives and Cancer Communication. J. Commun..

[30] Hoeken H., Kolthoff M., Sanders J. (2016). Story Perspective and Character Similarity as Drivers of Identification and Narrative Persuasion. Hum. Commun. Res..

[31] Loewenstein G.F. (2010). Insufficient Emotion: Soul-searching by a Former Indicter of Strong Emotions. Emot. Rev..

[32] Small D.A., Loewenstein G. (2003). Helping a Victim or Helping the Victim: Altruism and Identifiability. J. Risk Uncertain..

[33] Barraza J.A., Alexander V., Beavin L.E., Terris E.T., Zak P.J. (2015). The heart of the story: Peripheral physiology during narrative exposure predicts charitable giving. Biol. Psychol..

[34] Lin P.-Y., Grewal N.S., Morin C., Johnson W.D., Zak P.J. (2013). Oxytocin Increases the Influence of Public Service Advertisements. PLoS ONE.

[35] Moyer-Gusé E. (2008). Toward a Theory of Entertainment Persuasion: Explaining the Persuasive Effects of Entertainment Education Messages. Commun. Theory.

[36] Slater M.D., Rouner D. (2002). Entertainmen-Education and Elaboration Likelihood: Understanding the Processing of Narrative Persuasion. Commun. Theory.

[37] Green M.C., Brock T.C., Green M.C., Strange J.J., Brock T.C. (2002). In the Mind’s Eye Transportation-Imagery Model of Narrative Persuasion. Narrative Impact: Social and Cognitive Foundations.

[38] Graesser A.C., Olde B., Klettke B., Green M.C., Strange J.J., Brock T.C. (2002). How does the mind construct and represent stories?. Narrative Impact: Social and Cognitive Foundations.

[39] Graesser A.C., Ottati V., Wyer Robert S.J. (1995). Why Stories? Some evidence, questions, and challenges. Knowledge and Memory: The Real Story.

[40] Glaser M., Garsoffky B., Schwan S. (2009). Narrative-based learning: Possible benefits and problems. Commun. Med..

[41] Do Carmo Galiazzi M., Paula Salomão de Freitas D., Aguiar de Lima C., da Silva Cousin C., Langoni de Souza M., Launikas Cupelli R. (2019). Narratives of learning communities in environmental education. Environ. Educ. Res..

[42] Ge X., Shi X. (2014). Educational Narrative Inquiry in last decade: Changes of Methodology and Self-Consciousness. Educ. Res. Mon..

[43] Chapter 7 Science and Technology: Public Attitudes and Understanding. https://www.nsf.gov/statistics/seind14/content/chapter-7/chapter-7.pdf.

[44] Keller C., Siegrist M., Gutscher H. (2006). The Role of the Affect and Availability Heuristics in Risk Communication. Risk Anal..

[45] Rillo T.J. (1974). Basic guidelines for environmental education. J. Environ. Educ..

[46] Roth C.E. (1992). Environmental Literacy: It’s Roots Evolution and Directions in the 1990s.

[47] Dunlap R.E., Van Liere K.D. (1987). The New Environmental Paradigm: A Proposed Measuring Instrument and Preliminary Results. J. Environ. Educ..

[48] Weigel R.H., Weigel J. (1978). Environmental concern: The development of a measure. Environ. Behav..

[49] Dunlap R.E., Jones R.E., Dunlap R.E., William M. (2002). Environmental Concern. Handbook of Environmental Sociology.

[50] Wang M. (1999). Research on Environmental Awareness and Assessment Methods.

[51] Hong D. (1998). A Comprehensive Judge and Sample Analysis of the Citizens’ Environmental Awareness. Sci. Technol. Rev..

[52] Diekmann A., Preisendörfer P. (2001). Umweltsoziologie.

[53] Maloney M.P., Ward M.P. (1973). Ecology: Let’s hear from the people: An objective scale for the measurement of ecological attitudes and knowledge. Am. Psychol..

[54] Maloney M.P., Ward M.P., Braucht G.N. (1975). A revised scale for the measurement of ecological attitudes and knowledge. Am. Psychol..

[55] Catton W.R., Dunlap R.E. (1978). Environmental Sociology: A New Paradigm. Am. Sociol..

[56] Dunlap R.E., Liere K.D.V., Mertig A.G., Jones R.E. (2000). New Trends in Measuring Environmental Attitudes: Measuring Endorsement of the New Ecological Paradigm: A Revised NEP Scale. J. Soc. Issues.

[57] Urban D. (1986). Was ist Umweltbewußtsein? Exploration eines mehrdimensionalen Einstellungskonstruktes. Z. Soziologie.

[58] Gifford R., Nilsson A. (2014). Personal and social factors that influence pro-environmental concern and behaviour: A review. Int. J. Psychol..

[59] Hong D., Fan Y. (2016). Measuring Public Environmental Knowledge: The Development of An Indigenous Instrument and Its Assessment. J. Renmin Univ. China.

[60] Stern P., Dietz T., Guagnano G. (1995). The New Ecological Paradigm in Social-Psychological Context. Environ. Behav..

[61] Zhang H. (2015). Statistical Methods in Education and Psychology.

[62] Gunasekaran D.R. (2020). The Impact of Multimedia on Environmental Education Teaching at B.Ed., Level. Int. J. Trend Sci. Res. Dev..

[63] Sellmann D., Bogner F.X. (2013). Climate change education: Quantitatively assessing the impact of a botanical garden as an informal learning environment. Environ. Educ. Res..

[64] Stern M.J., Powell R.B., Ardoin N.M. (2008). What Difference Does It Make? Assessing Outcomes from Participation in a Residential Environmental Education Program. J. Environ. Educ..

[65] Liefländer A.K., Bogner F.X., Kibbe A., Kaiser F.G. (2015). Evaluating Environmental Knowledge Dimension Convergence to Assess Educational Programme Effectiveness. Int. J. Sci. Educ..

[66] Kelman H.C., Kelman H.C. (1965). Social-psychological approaches to the study of international relations: The question of relevance. International Behavior.

[67] Bogner F.X. (1998). The Influence of Short-Term Outdoor Ecology Education on Long-Term Variables of Environmental Perspective. J. Environ. Educ..

[68] Braun T., Dierkes P.W. (2017). Connecting students to nature-how the intensity of nature experience and student age influence the success of outdoor education programs. Environ. Educ. Res..

[69] Bandura A. (1977). Social learning theory. Can. J. Sociol. Cah. Can. De Sociol..

[70] Wilson M. (2002). Six views of embodied cognition. Psychon. Bull. Rev..

[71] Skinner’s Theory. http://psychlearning.com/skinners-theory.

